# Indoleamine 2,3-Dioxygenase Expression Pattern in the Tumor Microenvironment Predicts Clinical Outcome in Early Stage Cervical Cancer

**DOI:** 10.3389/fimmu.2018.01598

**Published:** 2018-07-11

**Authors:** A. Marijne Heeren, Ilse van Dijk, Daniella R. A. I. Berry, Maryam Khelil, Debbie Ferns, Jeroen Kole, René J. P. Musters, Victor L. Thijssen, Constantijne H. Mom, Gemma G. Kenter, Maaike C. G. Bleeker, Tanja D. de Gruijl, Ekaterina S. Jordanova

**Affiliations:** ^1^Center Gynecological Oncology Amsterdam (CGOA), Department of Obstetrics and Gynecology, VU University Medical Center, Amsterdam, Netherlands; ^2^Cancer Center Amsterdam, Departments of Medical Oncology & Radiation Oncology, VU University Medical Center, Amsterdam, Netherlands; ^3^Department of Pathology, VU University Medical Center, Amsterdam, Netherlands; ^4^Laboratory for Physiology, Institute for Cardiovascular Research, VU University Medical Center, Amsterdam, Netherlands; ^5^Center Gynecological Oncology Amsterdam (CGOA), Department of Obstetrics and Gynecology, Academic Medical Center, Amsterdam, Netherlands; ^6^Center Gynecological Oncology Amsterdam (CGOA), Department of Gynecology, Netherlands Cancer Institute – Antoni van Leeuwenhoek, Amsterdam, Netherlands

**Keywords:** cervical cancer, indoleamine 2,3-dioxygenase, kynurenine, tryptophan, serum, T cells, mRNA, The Cancer Genome Atlas

## Abstract

The indoleamine 2,3-dioxygenase (IDO) enzyme can act as an immunoregulator by inhibiting T cell function *via* the degradation of the essential amino acid tryptophan (trp) into kynurenine (kyn) and its derivates. The kyn/trp ratio in serum is a prognostic factor for cervical cancer patients; however, information about the relationship between serum levels and IDO expression in the tumor is lacking. IDO expression was studied in 71 primary and 14 paired metastatic cervical cancer samples by various immunohistochemical (IHC) techniques, including 7-color fluorescent multiparameter IHC, and the link between the concentration of IDO metabolites in serum, clinicopathological characteristics, and the presence of (proliferating) T cells (CD8, Ki67, and FoxP3) was examined. In addition, we compared the relationships between *IDO1* and *IFNG* gene expression and clinical parameters using RNAseq data from 144 cervical tumor samples published by The Cancer Genome Atlas (TCGA). Here, we demonstrate that patchy tumor IDO expression is associated with an increased systemic kyn/trp ratio in cervical cancer (*P* = 0.009), whereas marginal tumor expression at the interface with the stroma is linked to improved disease-free (DFS) (*P* = 0.017) and disease-specific survival (*P* = 0.043). The latter may be related to T cell infiltration and localized IFNγ release inducing IDO expression. Indeed, TCGA analysis of 144 cervical tumor samples revealed a strong and positive correlation between *IDO1* and *IFNG* mRNA expression levels (*P* < 0.001) and a significant association with improved DFS for high *IDO1* and *IFNG* transcript levels (*P* = 0.031). Unexpectedly, IDO+ tumors had higher CD8^+^Ki67^+^ T cell rates (*P* = 0.004). Our data thus indicate that the serum kyn/trp ratio and IDO expression in primary tumor samples are not clear-cut biomarkers for prognosis and stratification of patients with early stage cervical cancer for clinical trials implementing IDO inhibitors. Rather, a marginal IDO expression pattern in the tumor dominantly predicts favorable outcome, which might be related to IFNγ release in the cervical tumor microenvironment.

## Introduction

In cervical cancer, a persistent infection with high-risk human papillomavirus strains (mainly types 16 and -18) is responsible for initiating carcinogenesis (1). Expression of the viral E6 and E7 oncogenes is instrumental in this process, and thereby, cervical cancer is a relatively immunogenic disease, employing various escape mechanisms to avoid the host’s immune attack (2).

One of these putative tumor escape mechanisms is the expression of indoleamine 2,3-dioxygenase (IDO), which might be induced by IFNγ secretion by cytotoxic CD8^+^ T cells in the tumor microenvironment (3, 4). IDO is an intracellular enzyme that is able to catabolize tryptophan along the kynurenine pathway. Tryptophan is an essential amino acid, necessary for protein synthesis and other metabolic cell functions. Contradictory results have been reported about the actual effect of tryptophan depletion (5). Mostly, *in vitro* and *in vivo* mice studies have shown that particularly activated, not resting, T- and natural killer (NK) cells seem to be sensitive to tryptophan-depletion and the presence of kynurenine and its derivates in the microenvironment (6–11). Upon tryptophan depletion, arrest of the cell cycle takes place in the G-phase, which in turn renders T cells more sensitive to apoptosis (6, 12, 13). In addition, it has been shown that IDO-expressing tumors promote differentiation and activation of regulatory T cells (Tregs) (9, 14, 15), which in turn can induce IDO expression in myeloid cells *via* cytotoxic T-lymphocyte-associated protein-4 (CTLA-4)–CD80/86 interactions (16) and recruit myeloid-derived suppressor cells (MDSCs) to the tumor site (17, 18). Whereas the majority of reports point to a detrimental effect of IDO expression and activity on patient outcome in various tumor types (19), others have shown IDO to be associated with favorable outcome (20–26).

In cervical cancer, IDO expression has been observed in primary and metastatic tumor cells and in immune cells, like macrophages, dendritic cells, and NK cells (27–30). In addition, IDO activity, measured by the kynurenine/tryptophan (kyn/trp) ratio, in cervical cancer patients’ pretreatment sera has been reported by us and by others to be linked to disease stage and poor prognosis (31, 32). Currently, clinical trials in various tumor types are performed to explore the implementation of IDO inhibitors for cancer therapy (19), but to our knowledge, this does not yet include cervical cancer patients.

Here, for the first time, we searched for a link between IDO expression patterns in the tumor microenvironment and the presence of systemic IDO metabolites in early stage squamous cervical cancer. To this end, we have examined the association between IDO expression patterns in formalin-fixed, paraffin-embedded (FFPE) tumor tissue and the concentrations of IDO metabolites in serum. In addition, we studied the association of IDO expression patterns with clinicopathological features and the presence of proliferating cytotoxic CD8^+^ T cells and Tregs. Also, we compared the relationships between *IDO1* and *IFNG* gene expression and linked this to survival outcome using RNAseq data from cervical tumor samples published by The Cancer Genome Atlas (TCGA).

Our findings may contribute to the development of predictive biomarkers for clinical trials using IDO inhibitors and to the development of new and more effective immunotherapy strategies for cervical cancer.

## Materials and Methods

### Patient Cohort

Previously, we reported on the measurement of serum levels of IDO metabolites (tryptophan, kynurenine, and 3-hydroxykynurenine) in 251 cervical cancer patients (32). From this cohort, we selected all squamous cell carcinoma patients, diagnosed between 2003 and 2008, with surgery as primary treatment and with sufficient FFPE material available for our study. FFPE tissue blocks with 71 primary tumors (PTs) and 14 paired metastatic lymph nodes were obtained from the archives of the Department of Pathology at the Academic Medical Center (AMC) Amsterdam, The Netherlands. The main clinicopathological features of these patients are summarized in Table 1. None of the patients underwent chemotherapy or radiotherapy before surgery. The specimens were anonymously processed and selection of blocks was guided by initial diagnosis and review by the pathologist. Ethical approval was waived according to the regulations in The Netherlands (33).

**Table 1:** Patient distribution according to indoleamine 2,3-dioxygenase (IDO) expression in relation to clinicopathological characteristics.

			Tumor cells IDO expression			Tumor cells IDO expression pattern				Tumor-infiltrating immune cells			Stromal immune cells			Tumor-associated vessels		
		
Clinicopathological characteristics		Total *n* (%)	IDO− *n* (%)	IDO+ *n* (%)	*P*	Patchy *n* (%)	Patchy + margin *n* (%)	Margin *n* (%)	*P*	IDO− *n* (%)	IDO+ *n* (%)	*P*	IDO− *n* (%)	IDO+ *n* (%)	*P*	IDO− *n* (%)	IDO+ *n* (%)	*P*
Number of patients		71 (100)	15 (21)	56 (79)	–	33 (63)	14 (26)	6 (11)	*–*	33 (54)	28 (46)	*–*	7 (10)	61 (90)	*–*	60 (87)	9 (13)	*–*
Age in years*		44.9	39.0	46.5	***0.010***	45.6	49.4	44.8	*0.499*	43.3	48.1	*0.116*	44.7	45.5	*0.911*	44.8	48.4	*0.276*
FIGO stage^#^	IBI	55 (77)	10 (14)	45 (63)	*0.260*	25 (47)	11 (21)	6 (11)	*0.535*	23 (38)	24 (39)	*0.222*	4 (6)	50 (74)	*0.147*	48 (70)	6 (9)	*0.396*
	≥IBII	16 (23)	5 (7)	11 (16)		8 (15)	3 (6)	0 (0)		10 (16)	4 (7)		3 (4)	11 (16)		12 (17)	3 (4)	
Tumor size^#^^,^^a^	≤4 cm	59 (84)	10 (14)	49 (70)	***0.034***	28 (54)	13 (25)	6 (12)	*1.000*	27 (45)	23 (38)	*1.000*	6 (9)	51 (76)	*1.000*	51 (75)	7 (10)	*0.611*
	>4 cm	11 (16)	5 (7)	6 (9)		4 (8)	1 (2)	0 (0)		6 (10)	4 (7)		1 (2)	9 (13)		8 (12)	2 (3)	
Parametrium invasion^#^	No	57 (80)	13 (18)	44 (62)	*0.719*	26 (49)	10 (19)	5 (9)	*0.886*	25 (41)	22 (36)	*0.795*	5 (7)	50 (74)	*0.611*	53 (77)	3 (4)	***0.001***
	Yes	14 (20)	2 (3)	12 (17)		7 (13)	4 (8)	1 (2)		8 (13)	6 (10)		2 (3)	11 (16)		7 (10)	6 (9)	
Vaginal involvement^#^	No	65 (91)	13 (18)	52 (73)	*0.600*	31 (58)	12 (23)	6 (11)	*0.739*	28 (46)	27 (44)	*0.205*	5 (7)	57 (84)	*0.112*	56 (81)	7 (10)	*0.172*
	Yes	6 (9)	2 (3)	4 (6)		2 (4)	2 (4)	0 (0)		5 (8)	1 (2)		2 (3)	4 (6)		4 (6)	2 (3)	
Lymph node metastases	No	49 (69)	10 (14)	39 (55)	*0.825*	22 (42)	9 (17)	5 (9)	*0.824*	18 (30)	24 (39)	***0.012***	4 (6)	45 (66)	*0.390*	43 (62)	6 (9)	*0.712*
	Yes	22 (31)	5 (7)	17 (24)		11 (21)	5 (9)	1 (2)		15 (25)	4 (6)		3 (4)	16 (24)		17 (25)	3 (4)	

*FIGO: International Federation of Gynecology and Obstetrics*.

*^a^Of one case we do not have information on tumor size*.

*^#^P-value measured with Asymptotic Pearson’s and Fisher’s exact test was used when counts were <5*.

*P-value was calculated with Mann–Whitney *U* test. IDO+, IDO-positive; IDO−, IDO-negative. NB: in three cases, we found it difficult to score IDO+ tumors for their expression pattern (patchy/margin) due to small tumor fields. In some cases (*n* = 10), we found it difficult to distinguish between IDO-positive immune cells and IDO-positive tumor cells and excluded those cases for scoring expression in infiltrating cells. In some cases (*n* = 3), we found the staining pattern not convincing due to small stromal fields in the stained tissue section and excluded those cases for scoring stromal IDO+ cells. In some cases (*n* = 2), we found it difficult to score IDO expression in vessels and excluded those cases.

*P value in bold italic is <0.05*.

P value in italic is >0.05

### Immunohistochemistry

Immunohistochemical staining of 71 PTs and 14 metastatic lymph nodes was performed as previously described (34) using Tris/EDTA buffer pH 9.0 for antigen retrieval, mouse-IgG1 anti-IDO antibody (1F8.2, Millipore), and ready to use Poly-HRP-GAM/R/R IgG (ImmunoLogic, The Netherlands). Complexes were visualized using 3,3′-diaminobenzidine tetrahydrochloride (Sigma, USA). Slides were counterstained with hematoxylin.

### Multiplex Immunohistochemistry

On a representative subset of patients, quadruple immunofluorescence staining was performed as previously described (35) using Tris/EDTA buffer pH 9.0 for antigen retrieval. Primary antibodies mouse-IgG1 anti-FoxP3 (236A/E7; Abcam, UK), rabbit anti-Ki67 (SP6; ThermoFisher, USA), mouse-IgG2b anti-CD8 (4B11; Novocastra, UK), and secondary antibodies goat anti-mouse IgG1 Alexa Fluor 488, goat anti-Rabbit IgG Alexa Fluor 546, and goat anti-mouse IgG2b Alexa Fluor 647 (all from Thermo Scientific, USA) were used for T cell phenotyping (*n* = 35). Primary antibodies mouse-IgG1 anti-IDO (1F8.2, Millipore), mouse-IgG2a CD14 (clone 7, Abcam), rabbit anti-HLA-DR (ab137832, Abcam), and secondary antibodies goat anti-mouse IgG1 Alexa Fluor 488, goat anti-mouse IgG2a Alexa Fluor 546, and goat anti-Rabbit IgG Alexa Fluor 647 (all from Thermo Scientific, USA) were used for IDO-positive myeloid cell identification (*n* = 6). 4’,6-diamidino-2′-phenylindole, dihydrochloride (DAPI; Thermo Scientific, USA) was used as a counterstain, slides were enclosed with mounting medium and coverslips.

Multiplexed immunofluorescence staining was performed on eight patients in order to identify the type of tumor-associated vessels expressing IDO, using the OPAL 7-color fluorescence immunohistochemistry (IHC) Kit (Perkin Elmer, USA). A blocking step for endogenous peroxidase was introduced with 0.3% H_2_O_2_/methanol for 20 min and an extra fixation step was included for 20 min with 10% neutral buffered formalin (Leica Biosystems, Germany), followed by 2 min in Milli-Q water and 2 min in 0.05% Tween20 in 1× Tris-buffered saline (TBST). The following primary antibodies were used: mouse-IgG1 anti-CD34 (QBEND-10; Cell Sciences), mouse-IgG2a anti-α-smooth muscle (α-sma) actin (1A4; DAKO), mouse-IgG1 anti-CD31 (JC70A; DAKO), mouse-IgG1 anti-IDO (1F8.2, Millipore), mouse-IgG1 anti-podoplanin (D2-40; BIO-RAD), and rabbit anti-galectin-1 (500-P210, PeproTech). Steps were repeated for each primary antibody; microwave treatments were carried out by placing the slides in a plastic tray, after which they were heated in 0.05% ProClin300/Tris-EDTA buffer at pH 9.0 in an 800 W standard microwave at 100% power until boiling point, followed by 15 min at 30% power. Slides were cooled down in ice water, washed in Milli-Q water and in 1× TBST, and were blocked with Normal Antibody Diluent (Immunologic, The Netherlands) for 10 min at room temperature (RT). After that, slides were incubated with primary antibody diluted in Normal Antibody Diluent for 30 min at RT and 30 rounds per minute (rpm) on a shaker. Next, slides were washed 3 × 2 min in 1× TBST at RT and 30 rpm and were subsequently incubated with SuperPicture Polymer Detection Kit—HRP—broad spectrum (Life Technologies, USA) for 20 min at RT and 30 rpm. Afterward, slides were washed 3 × 2 min in 1× TBST and were incubated with Opal fluorochromes (Opal520, Opal570, Opal650, Opal690 Opal540, and Opal620) diluted 1:150 in amplification buffer (all provided by the OPAL 7-color fluorescence IHC Kit) for 10 min at RT and 30 rpm. Slides were then washed 3 × 2 min in TBST. Finally, microwave treatment with AR6 buffer was performed and slides were washed for 2 min in Milli-Q water and for 2 min in TSBT. DAPI working solution (provided by the OPAL 7-color fluorescence IHC Kit) was applied for 5 min at RT and the slides were washed again in Milli-Q water and in 1× TSBT, and then mounted under coverslips with ProLong Diamond antifade mounting medium (Life Technologies, USA).

### Imaging and Scoring

The standard IHC stained sections were scored for IDO expression by tumor cells, immune cells, and tumor-associated vessels using an Olympus BX50 bright-field microscope (Olympus, USA) by two investigators (A. Marijne Heeren and Ekaterina S. Jordanova). Primary- and metastatic tumors were designated IDO-negative (<1% of tumor cells expressed IDO) or IDO-positive (≥1% of the tumor cells expressed IDO). Also, tumor cells were divided in different groups: 0, 1–10, 10–50, and >50% positive for IDO, also used by others (27, 30). Furthermore, a distinction was made between patchy (patchy IDO expression throughout the whole tumor field) or marginal (focal staining, on the border between tumor and stroma) expression by tumors. Also, the presence of IDO-expressing tumor-infiltrating immune cells was scored in primary- and metastatic tumor samples either as absent (−) or present (+). Furthermore, IDO expression by stromal immune cells was scored as either present in low numbers (−) or high numbers (+) in stroma of PTs. In the metastatic lymph nodes, scores for IDO-positive immune cells were obtained, high (+) or low (−) numbers, for peritumoral area or in resident lymph node tissue distant from the tumor metastases. Finally, IDO expression by tumor-associated vessels was scored as 0/a few IDO-positive vessels (−) or all vessels positive for IDO (+).

Quadruple immunofluorescence stainings were imaged and analyzed using a digital imaging fluorescence microscope (Axiovert-200M; Zeiss, Germany) and SlideBook 6 Reader [Intelligent Imaging Innovations (3I), USA]. DAPI staining was used to morphologically distinguish tumor fields from stromal and healthy tissue. From each PT, three to five representative areas, including both tumor and stroma, were randomly selected and imaged with a 20× dry objective with 0.3 numerical aperture (NA). CD8-positive, FoxP3-positive, and Ki67-positive cells from digital images were manually enumerated and results were expressed as number of positive cells per square millimeters.

Seven-color multiplex stainings were visualized with Leica TCS SP8 microscope (Leica, Germany), tilescan (3 × 3, 40× oil objective with 1.3 NA) images were generated and viewed using LAS AF Lite software (Leica, Germany). IDO-positive tumor-associated vessels were analyzed for colocalization with the markers CD34, podoplanin, α-sma, galectin-1, and CD31.

### TCGA RNAseq Patient Cohort

Level 3 RSEM normalized, log-transformed RNAseq data, profiled using the Illumina HiSeq RNAseq v2, were retrieved from the TCGA data portal (36). Results of the TCGA RNAseq analysis have been described in detail by TCGA Research Network (37). For our analysis, data on 144 cervical SCC patients were used, including downloaded survival data (38), *IDO1* mRNA, and *IFNG* mRNA expression in PT samples.

### Statistical Analysis

Statistical analyses were performed using IBM SPSS (IBM, USA) and GraphPad Prism 5 (GraphPad Software, USA) software. Associations between IDO expression patterns in the tumor microenvironment and serum concentrations were performed using the same cutoff “low” (quartiles 1–3) and “high” (quartile 4) as previously been ascribed for survival analysis (32). Fisher’s exact test was used to study the association between IDO expression in the tumor microenvironment and serum concentrations of IDO metabolites for (sub)groups with three or more patients. The Mann–Whitney *U* test, Asymptotic Pearson’s- or Fisher’s exact tests were used for the comparison of IDO expression patterns and clinicopathological characteristics. The log-rank test was performed for survival analyses. Before association, analyses between local IDO protein expression and T cells were carried out, normal distribution was tested using the D’Agostino and Pearson omnibus normality test. Then, based on the observed distribution, Mann–Whitney *U* test or unpaired *t* test were used or the Kruskal–Wallis or one-way ANOVA, with *post hoc* Dunn’s Multiple Comparison or Bonferroni’s Multiple comparison tests, respectively. Furthermore, correlation analysis between *IDO1* and *IFNG* mRNA levels, retrieved form the TCGA database, was performed by Pearson’s correlation. Hierarchical cluster analysis was carried out using Euclidean distance and Ward.D2 clustering methods with the function heatmap.plus in RStudio Version 1.1.423 (RStudio, USA). Survival analysis for *IDO1* mRNA and *IFNG* mRNA were performed using the two acquired clusters (low and high) or using the median as cutoff (low and high).

Comparisons and associations with *P*-values below 0.05 were considered statistically significantly different.

## Results

### IDO Expression in PTs

**Figure 1:**
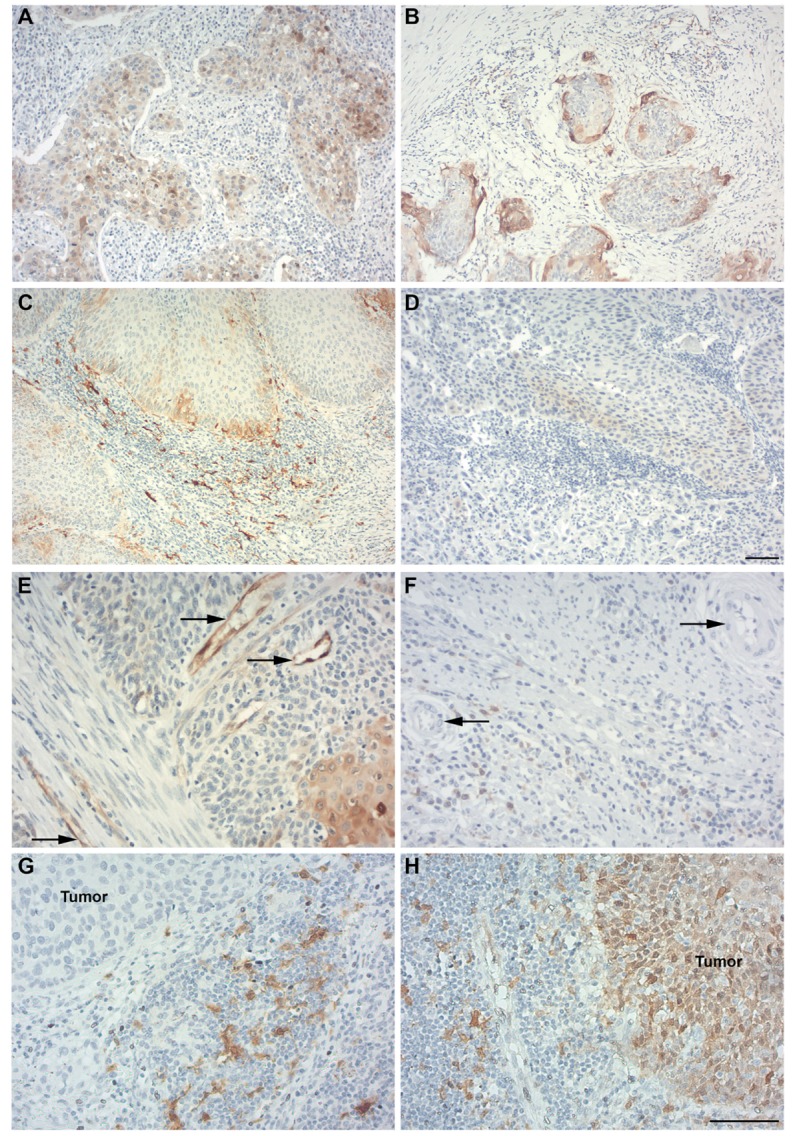
Indoleamine 2,3-dioxygenase (IDO) expression in primary cervical cancer and metastatic lymph nodes. Different patterns for IDO expression (in brown) were detected in primary- and metastatic cervical squamous cell carcinoma. **(A)** Patchy pattern with cytoplasmic IDO expression by primary tumor (PT) cells. **(B)** Marginal IDO expression by PT cells. **(C)** High numbers of IDO-expressing stromal immune cells in a marginal IDO-expressing tumor. **(D)** IDO-negative tumors, with low IDO expression in PT cells and stromal immune cells. **(E)** IDO-positive tumor-associated vessels (indicated by black arrows). **(F)** IDO-negative tumor-associated vessels (indicated by black arrows). **(G)** Metastatic lymph node sample showing metastatic tumor cells negative for IDO and IDO-positive immune cells surrounding the tumor fields. **(H)** Metastatic lymph node sample showing nuclear and cytoplasmic IDO expression by metastatic tumor cells and IDO-positive immune cells. Magnification for **(A–D)** is 100× [scale bar in **(D)** is 100 µm] and for **(E–H)** [scale bar in **(H)** is 100 µm] is 200×.

Indoleamine 2,3-dioxygenase protein expression was analyzed by immunohistochemistry. In the PT samples, we observed IDO positivity in tumor cells in a patchy and/or marginal expression pattern. Also, IDO expression was seen in tumor-infiltrating immune cells, stromal immune cells, and tumor-associated vessels. Among patients, various heterogeneous IDO expression patterns were observed (Figures 1A–F for representative images and Table S1 in Supplementary Material for IDO IHC scores per patient).

**Figure 2:**
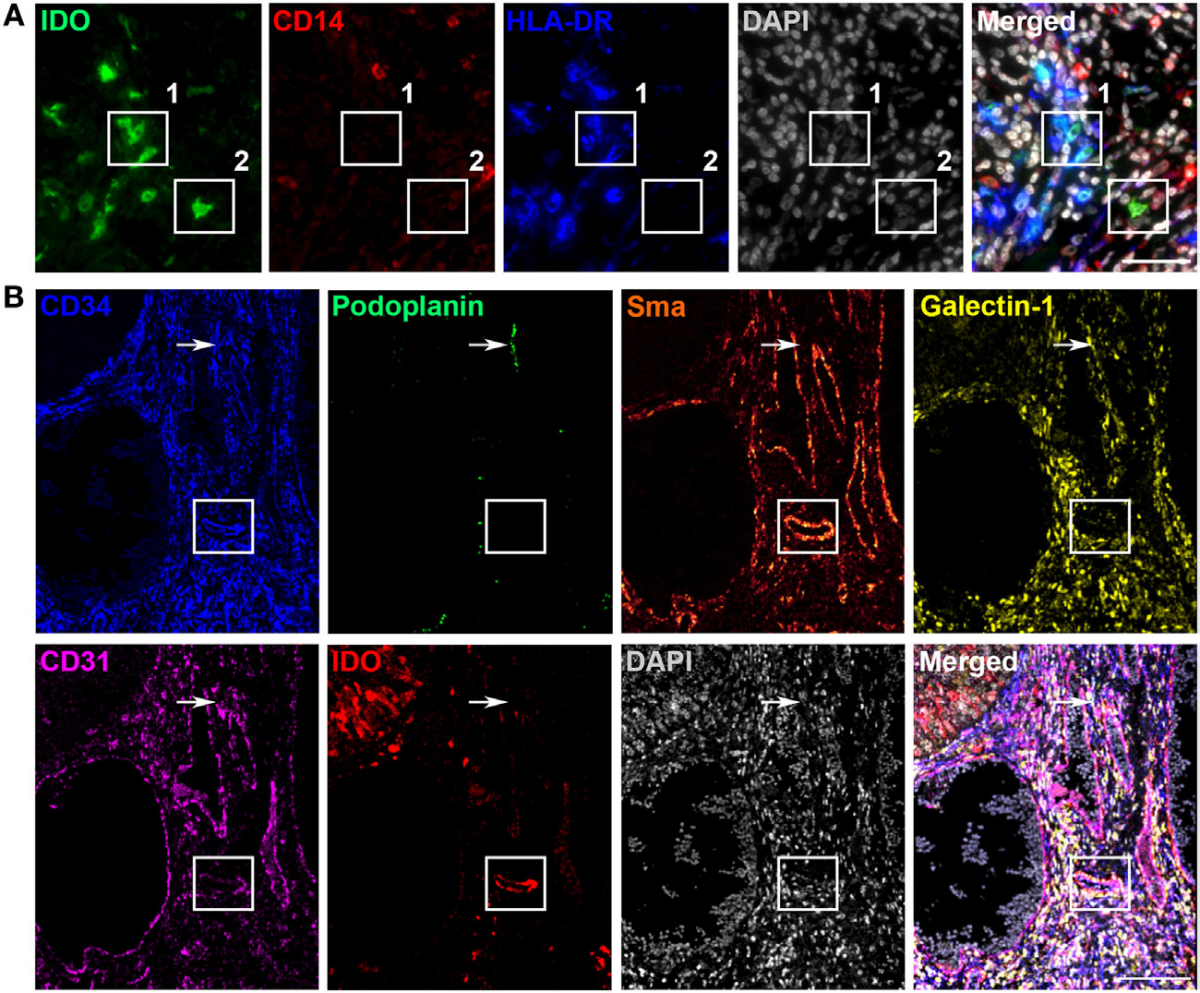
Characterization of indoleamine 2,3-dioxygenase (IDO)-positive immune cells and tumor-associated vessels in primary cervical cancer. Different immune cells expressing IDO were detected in primary cervical cancer. **(A)** Representative immunofluorescence images showing monochromatic IDO (in green), CD14 (in red), HLA-DR (in blue), DAPI (in gray), and the merged panel with IDO, CD14, HLA-DR, and DAPI in the stromal compartment. Box 1 shows IDO^+^CD14^−^HLA-DR^+^ cells and box 2 shows an IDO^+^CD14^−^HLA-DR^−^ cell. Scale bar is 100 µm. **(B)** Representative immunofluorescence tilescan of a IDO-positive tumor showing monochromatic CD34 (in blue), podoplanin (in green), α-sma (in glow), galectin-1 (in yellow), CD31 (in pink), IDO (in red), DAPI (in gray), and the merged image. Box indicates an IDO-positive vessel expressing CD34, α-sma, galectin-1, and CD31. Arrow indicates an IDO-negative podoplanin-positive lymphatic vessel. Scale bar is 95 µm.

Next, we aimed to further delineate the specific cell subpopulations and vessel types expressing IDO. We hypothesized that most IDO-positive immune cells were monocytic MDSCs or tumor-associated macrophages and tried to identify these cells using multicolor fluorescent immunohistochemistry for IDO, CD14, and HLA-DR in PT section from six cervical cancer patients. IDO-positive tumor- and stroma-infiltrating cells represented a heterogeneous population of immune cells consisting of HLA-DR^−^CD14^+^IDO^+^ MDSC-like cells, HLA-DR^+^CD14^+^IDO^+^ dendritic/macrophage-like cells, HLA-DR^−^CD14^−^IDO^+^-, and HLA-DR^+^CD14^−^IDO^+^ cells (Figure 2A). IDO-positive tumor-associated vessels were studied by 7-color multiplex immunohistochemistry using the markers CD31/CD34 (endothelial cell markers), podoplanin (lymphatic endothelial cell marker), α-sma (perivascular cell marker), galectin-1 (activated endothelial cell marker), and IDO. The IDO-positive tumor-associated vessels were predominantly identified as mature blood vessels since most vessels stained positive for α-sma, a marker of pericytes that cover mature vessels (Figure 2B). In two patients, IDO expression was also observed in lymphatic (podoplanin-positive) vessels.

### IDO Expression in Metastatic Lymph Nodes

In the 14 metastatic lymph node specimens available, we observed IDO positivity in tumor cells, tumor-infiltrating immune cells, immune cells surrounding metastatic tumor cells, and in resident lymph node tissue (Figures 1G,H). No IDO-positive vessels were observed. See Table S2 in Supplementary Material for an overview of the IDO IHC scores.

We found no evidence for elevated expression of IDO in the metastatic tumors as compared to the corresponding primary lesions. In one out of 14 metastatic lymph nodes, tumor cells were not detectable in the available tissue sections. In 8 out of 14 metastatic samples, IDO-positive tumor cells were detected. Interestingly, 7 out of 8 metastatic tumors showed a patchy IDO expression pattern. Paired analysis showed that six IDO patchy/patchy + margin expressing PTs retained patchy expression in the metastatic tumor cells, one marginal IDO-expressing PT had a patchy IDO-expressing metastatic tumor, one patchy IDO-expressing PT had a marginal IDO-expressing metastatic tumor, two IDO-negative PTs remained negative for IDO in the metastatic tumor cells and for three IDO-positive (patchy) PTs, corresponding metastatic tumors were negative for IDO (data not shown).

### Association Between IDO Expression at the Tumor Site and kyn/trp Ratio in Serum

To determine, in our patient cohort (*n* = 71), whether IDO-positivity in the PT microenvironment correlates with serum levels of IDO metabolites tryptophan, kynurenine, and 3-hydroxykynurenine, we used previously measured serum levels from a cohort of 251 cervical cancer patients where a high kyn/trp ratio was shown to be detrimental for survival (32). The interquartile concentrations of tryptophan, kynurenine, and 3-hydroxykynurenine and the kyn/trp ratio for the current patient cohort are summarized in Table 2 and were used for analysis.

**Table 2:** Concentration of indoleamine 2,3-dioxygenase metabolites in serum (*n* = 71).

	Q1	Q2	Q3	Q4
Tryptophan (μmol/L)	10.86–42.36	42.37–49.73	49.74–55.37	55.38–79.88
Kynurenine (μmol/L)	0.22–1.33	1.34–1.50	1.51–1.64	1.65–2.54
3-Hydroxykynurenine (nmol/L)	3.20–24.89	24.90–32.09	32.10–37.79	37.80–84.90
Kyn/Trp ratio	16.78–25.40	25.41–28.62	28.63–34.91	34.92–52.37

*Q, quartile*.

**Figure 3:**
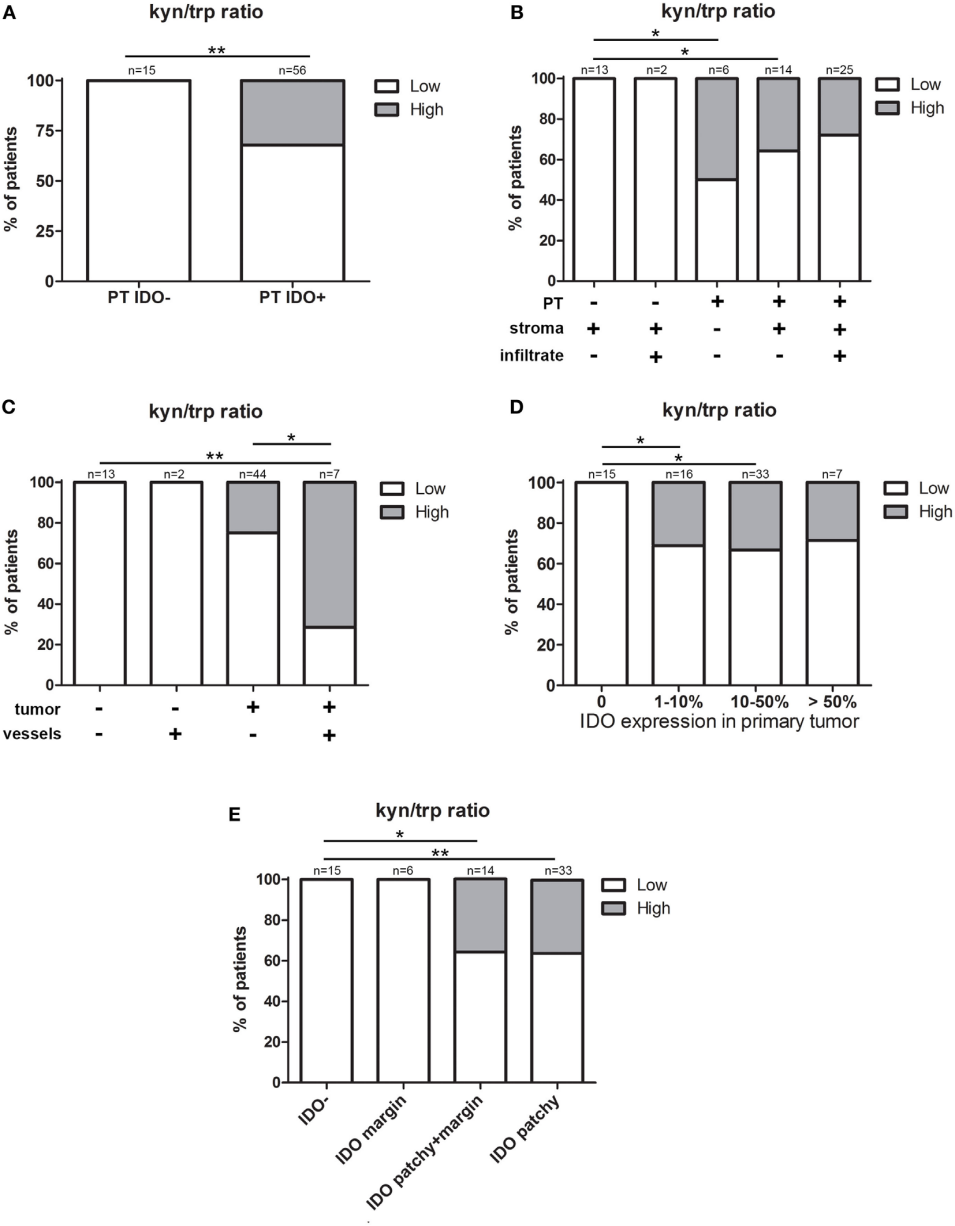
The influence of indoleamine 2,3-dioxygenase (IDO) expression in the local tumor microenvironment on the kyn/trp ratio in serum. Graphs show low (16.78–34.91, white) and high (34.92–52.37, gray) serum kynurenine/tryptophan (kyn/trp) ratio for **(A)** patients with IDO expression (IDO−/IDO+) in primary tumor (PT). Further stratification for expression patterns in **(B)** stroma and infiltrating immune cells (stroma and infiltrate) and **(C)** tumor-associated vessels (vessels). **(D)** Patients with IDO-negative tumors (0%) and patients with IDO-positive tumors divided into groups of 1–10, 10–50, and more than 50% of IDO positivity in tumor cells, and for **(E)** patient groups with different IDO expression patterns including IDO-negative (IDO−), IDO margin, IDO patchy + margin, and patchy IDO expression by PTs. *P* values were calculated excluding subgroups with *n* = 2 or smaller, using (pairwise) Fisher’s exact test. *P = 0.01–0.05 and **P = 0.01–0.001.

We analyzed whether IDO expression in the local tumor microenvironment influenced the levels of IDO metabolites in serum. Notably, we found a significant association between IDO positivity in the PT and a high kyn/trp ratio in serum (*P* = 0.008, Fisher’s exact test) (Figure 3A), independent of IDO expression by immune cells (infiltrate and stroma) (Figure 3B). Furthermore, patients with both IDO-positive tumors and vessels had significantly more often a high kyn/trp ratio in serum compared to patients with both IDO-negative tumors and vessels (*P* = 0.001, pairwise Fisher’s exact test) and patients with IDO-positive tumors and IDO-negative vessels (*P* = 0.025, pairwise Fisher’s exact test) (Figure 3C). Interestingly, we found that the dominance of tumor cell expression on systemic serum levels was independent of the percentage IDO-positive tumor cells (Figure 3D), but that the serum kyn/trp ratio was apparently determined by the different IDO expression patterns of the PT. All patients with IDO-negative tumors and marginal IDO-expressing tumors had a low kyn/trp ratio, whereas patients with patchy/patchy + marginal IDO expression had more often a high kyn/trp ratio in serum, which was significantly elevated when compared to patients with IDO-negative tumors (*P* = 0.009 and *P* = 0.017 respectively, pairwise Fisher’s exact test) (Figure 3E).

No associations were found for IDO positivity in the PT microenvironment and the individual IDO metabolites tryptophan, kynurenine, and 3-hydroxykynurenine in serum. Of note, the number of metastatic lymph nodes analyzed was too small for association analysis of IDO expression with serum kyn/trp levels.

### IDO Expression in Relation to Clinicopathological Characteristics and Survival

In Table 1, the associations between IDO expression patterns and clinicopathological characteristics of the patient cohort are shown.

**Figure 4:**
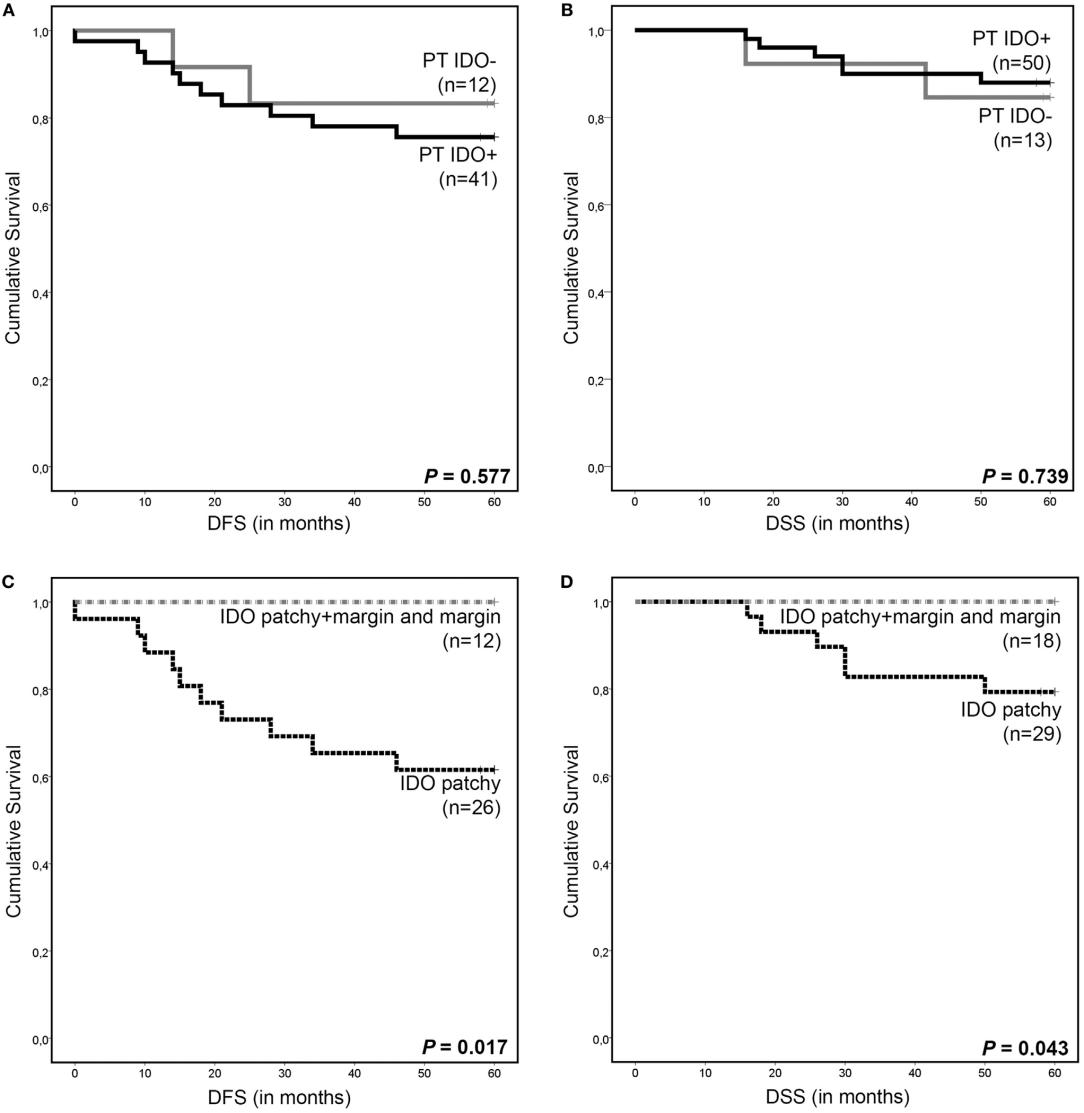
Survival analysis according to indoleamine 2,3-dioxygenase (IDO) expression in cervical cancer. Kaplan–Meier 5-year survival curve shows **(A)** disease-free survival (DFS) and **(B)** disease-specific survival (DSS) for patients with IDO-positive primary tumors (PT IDO+, black line) and IDO-negative (PTs IDO−, gray line). Kaplan–Meier 5-year survival curves show **(C)** DFS and **(D)** DSS for patients with patchy IDO expression (black dotted line), for patients with marginal, including patchy + marginal, IDO expression (gray dotted line) by PT cells. *P* values were calculated between the different groups using the log-rank test. NB: for some patients, DFS and DSS data are missing due to loss of follow-up.

Interestingly, patients with IDO-positive tumors were older (46.5 vs. 39 years old) (*P* = 0.010, Mann–Whitney *U* test) and manifested more often with smaller tumors (≤4 cm) (*P* = 0.034, Asymptotic Pearson’s χ^2^-test). However, no difference was observed in survival outcome between patients with IDO-negative and IDO-positive tumors *per se* (Figures 4A,B). Remarkably, all patients with marginal, including combined patchy + margin, IDO expression were disease free and still alive after a median follow-up of 60 months. These patients had improved disease-free survival (DFS) (*P* = 0.017, log-rank test) and disease-specific survival (DSS) (*P* = 0.043, log-rank test) as compared to patients with patchy IDO expression only (Figures 4C,D).

In addition, patients with IDO-positive tumor-infiltrating immune cells had less often lymph node metastases (*P* = 0.012, Fisher’s exact test). Interestingly, patients with IDO-negative tumor-associated vessels had less often parametrium invasion (*P* = 0.001, Fisher’s exact test). No further significant correlations were found. IDO expression in tumor-infiltrating immune cells and tumor-associated vessels did not affect survival (data not shown).

### IDO Expression in Relation to the Distribution and Localization of T Cells

**Figure 5:**
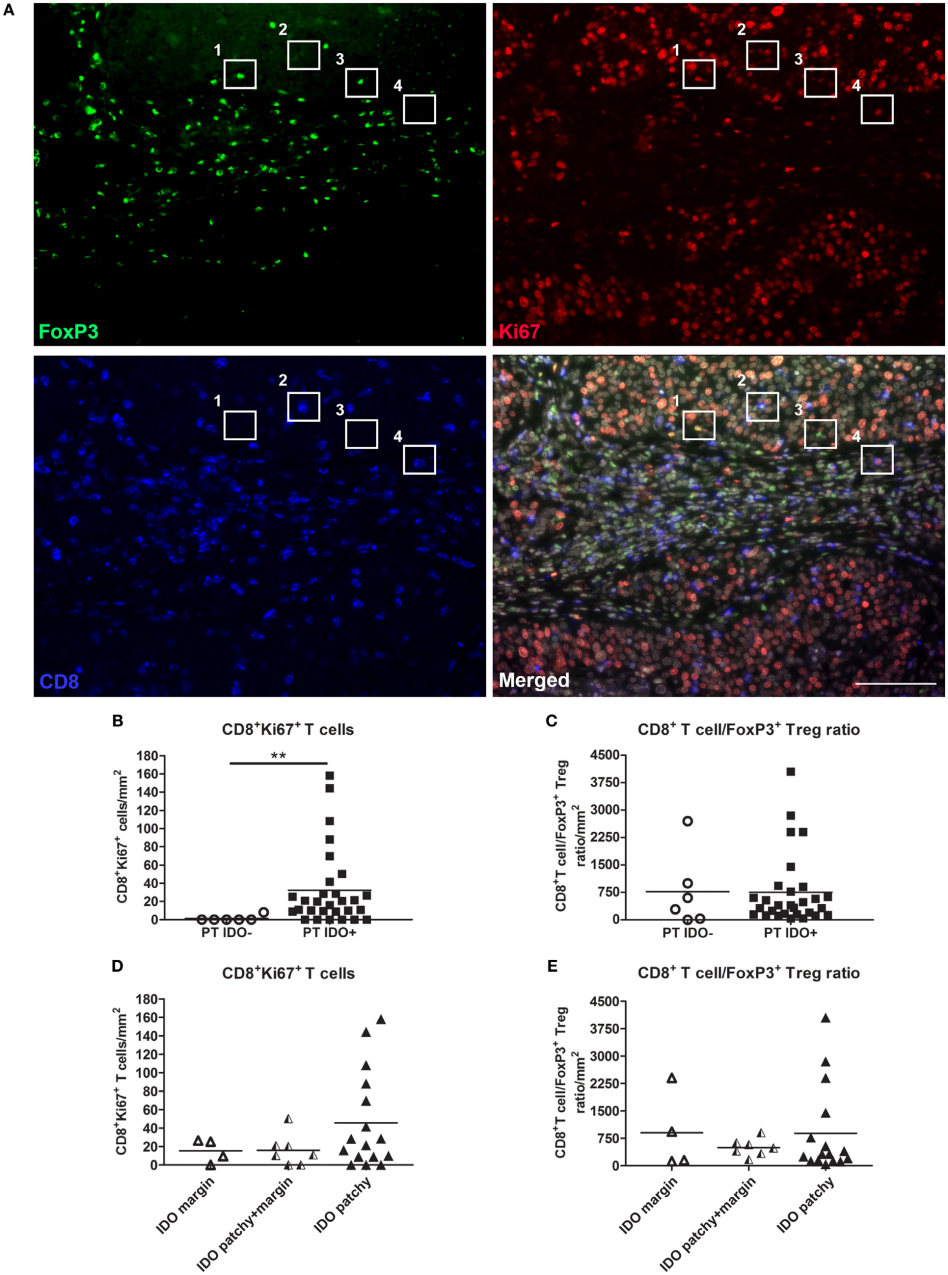
T cell numbers in relation to indoleamine 2,3-dioxygenase (IDO) expression by primary cervical tumor cells. **(A)** Representative immunofluorescence images showing monochromatic FoxP3 (in green), Ki67 (in red), CD8 (in blue), and the merged panel with FoxP3, Ki67, CD8 and DAPI. In box 1 a proliferating Ki67^+^FoxP3^+^ T cell is depicted, in box 2 a cytotoxic CD8^+^ T cells is depicted, in box 3 a FoxP3^+^ Treg is depicted, and in box 4 a proliferating CD8^+^Ki67^+^ T cell is depicted. Scale bar is 100 µm. Scatter plots show intratumoral numbers per square millimeters for **(B)** CD8^+^Ki67^+^ T cells and **(C)** CD8^+^ T cell/FoxP3^+^ Treg ratio in IDO-negative (*n* = 6, white dots) and IDO-positive (*n* = 29, black squares) primary tumors (PTs). Scatter plots show intratumoral numbers per square millimeters for **(D)** CD8^+^Ki67^+^ T cells and **(E)** CD8^+^ T cell/FoxP3^+^ Treg ratio in PTs with marginal IDO (*n* = 4, white triangles), patchy + marginal IDO (*n* = 7, black/white triangles), and patchy IDO expression (*n* = 16, black triangles). *P* values were calculated using Mann–Whitney *U* test. **P = 0.004.

Next, in order to study the effect of IDO expression on tumor-infiltrating T cell numbers, we quantified cytotoxic CD8^+^ T cells, FoxP3^+^(CD8^−^) Tregs, proliferating CD8^+^Ki67^+^ T cells, proliferating FoxP3^+^Ki67^+^(CD8^−^) T cells (proliferative Tregs), and FoxP3^+^CD8^+^ T cells per square millimeters in a representative subset of patients (*n* = 35) (Figure 5A). Nuclear DAPI stain was used to distinguish tumor tissue from stromal tissue. Unexpectedly, we observed higher counts of intratumoral CD8^+^Ki67^+^ T cells in IDO-positive tumors as compared to IDO-negative tumors (*P* = 0.004, Mann–Whitney *U* test) (Figure 5B). No significant differences were found between IDO-negative- and IDO-positive tumors for any of the other T cell subtypes (Figure 5C and data not shown). Also, the IDO expression patterns, marginal, patchy + marginal, or patchy did not affect infiltrating T cell numbers (Figures 5D,E and data not shown).

Furthermore, we observed higher rates of intratumoral cytotoxic CD8^+^ T cells (*P* = 0.041, Mann–Whitney *U* test), a higher intratumoral CD8^+^ T cell/FoxP3^+^ Treg ratio (*P* = 0.012, unpaired *t* test), higher rates of CD8^+^Ki67^+^ T cells both in the stromal (*P* = 0.004, Mann*–*Whitney *U* test) and intratumoral (*P* < 0.001, Mann–Whitney *U* test) compartment, in tumors with IDO-positive tumor-infiltrating immune cells (Figures S1A–C in Supplementary Material). Significantly higher rates of intratumoral CD8^+^Ki67^+^ T cells were observed in total IDO-positive PTs (PT+stroma+infiltrate+) vs. partly IDO-positive PTs (PT+stroma+infiltrate−) (Figure S1D in Supplementary Material, both *P* < 0.01). No further significant associations were found.

### *IDO1* vs. *IFNG* mRNA Expression

**Figure 6:**
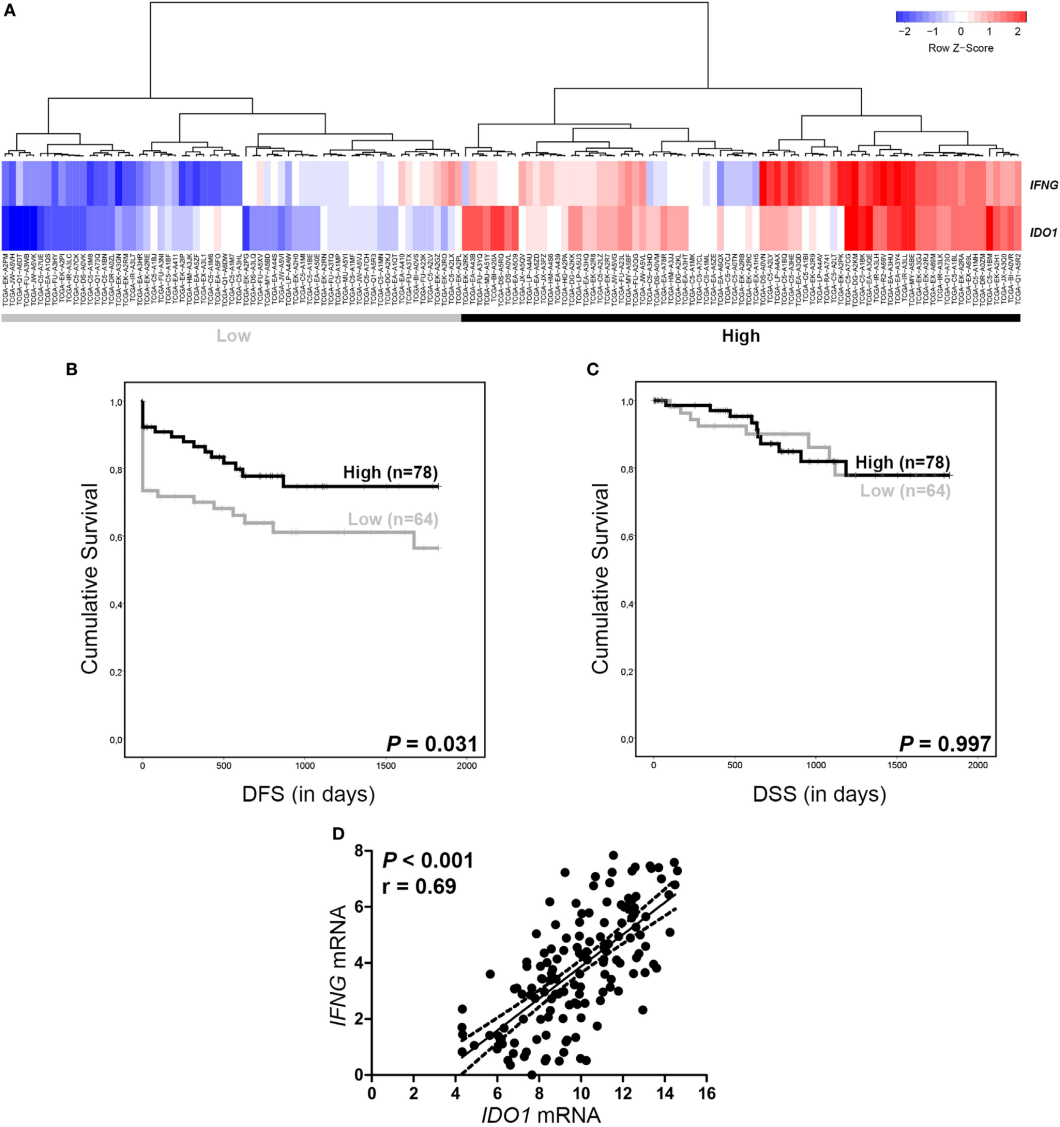
*IDO1* and *IFNG* RNAseq data from The Cancer Genome Atlas. **(A)** Hierarchical clustering of *IDO1* and *IFNG* mRNA (rows) measured on primary tumor samples from 144 cervical SCC patients (columns) reveals a “High” (for both *IDO1* and *IFNG*) and a “Low” (for both *IDO1* and *IFNG*) patient group. Kaplan–Meier 5-year survival curve shows **(B)** disease-free survival (DFS) and **(C)** disease-specific survival (DSS) for patients with both high *IDO1* and *IFNG* (black line) and patients with both low *IDO1* and *IFNG* (gray line), based on hierarchical cluster analysis. **(D)** Graph shows correlation between *IDO1* and *IFNG*. NB: survival data was missing for two patients. *P* values for survival analysis were calculated using the log-rank test. *P* value for correlation analysis was calculated using Pearson’s correlation.

To test whether RNAseq data of PT samples could be used for the validation of IDO protein expression and to study a possible link between IDO and IFNγ (39), we retrieved *IDO1* and *IFNG* gene expression data from 144 cervical SCC patients from TCGA Research Network database (37). Hierarchical clustering revealed two groups: patients with both low *IDO1* and *IFNG* mRNA expression (“Low” group) and patients with both high *IDO1* and *IFNG* mRNA expression (“High” group) (Figure 6A). For DSS analysis, no significant associations were found between the two patient groups (Figure 6C). However, DFS analysis showed an improved outcome for patients with “High” *IDO1* and *IFNG* as compared to patients with “Low” *IDO1* and *IFNG* mRNA expression (*P* = 0.031, log-rank test) (Figure 6B). Interestingly, *IDO1* and *IFNG* mRNA expression were strongly and significantly correlated (*P* < 0.001, Pearson’s correlation) (Figure 6D).

**Figure 7:**
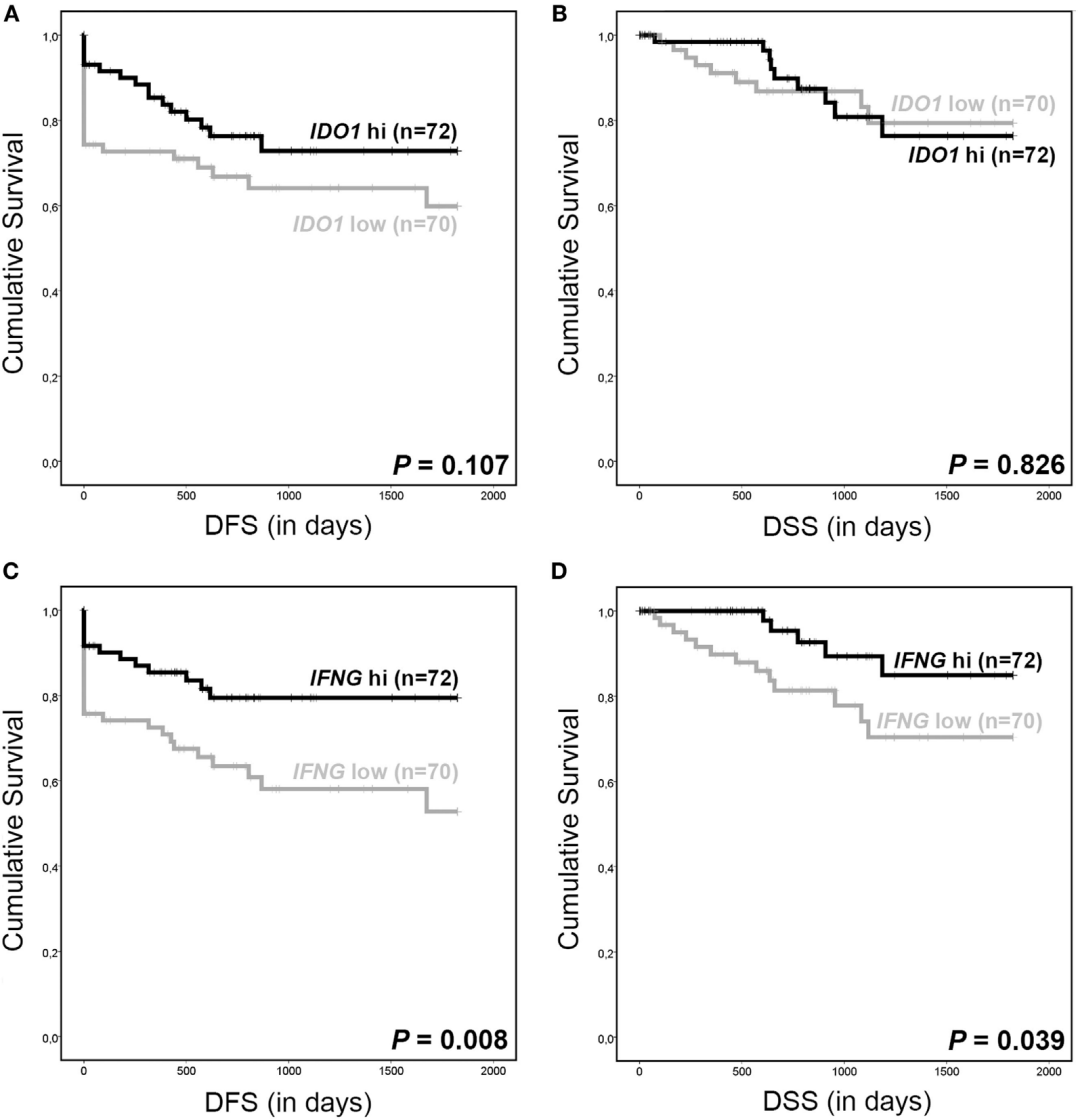
*IDO1* and *IFNG* RNAseq data from The Cancer Genome Atlas. Kaplan–Meier 5-year survival curve shows **(A)** disease-free survival (DFS) and **(B)** disease-specific survival (DSS) for patients with low (gray line, median as cutoff, <9.92) and high (black line, median as cutoff, ≥9.92) *IDO1* mRNA expression. Kaplan–Meier 5-year survival curve shows **(C)** DFS and **(D)** DSS for patients with low (gray line, median as cutoff, <3.71) and high (black line, median as cutoff, ≥3.71) *IFNG* mRNA expression. NB: survival data were missing for two patients. *P* values for survival analysis were calculated using the log-rank test.

Also, when TCGA tumors were divided into two groups based on above- or below-median *IDO1* mRNA (9.92) and *IFNG* mRNA (3.71) expression levels, *IDO1* mRNA expression was not linked to survival outcome (Figures 7A,B), whereas for patients with above median *IFNG* mRNA expression, an improved DFS (*P* = 0.008, log-rank test) and DSS (*P* = 0.039, log-rank test) was observed (Figures 7C,D).

## Discussion

Expression of the metabolic enzyme IDO is one of the many immune escape mechanisms employed by tumor cells (40). Many clinical trials have investigated, or are currently investigating, the effect of IDO inhibitors [i.e., Epacadostat and Indoximod (1-Methyl-d-Tryptophan)], and IDO peptide vaccination in cancer patients (19, 41–44). Currently, patients entering these clinical trials are not stratified for IDO positivity in tumor biopsies and/or systemic kyn/trp levels. Such information could be helpful in order to achieve higher immunotherapy response rates and avoid unnecessary over-treatment. Moreover, it was suggested that IDO activity in serum can be influenced by other factors such as chronic infection, neuropsychiatric diseases, and diet (45–48). Regrettably, extensive studies on the systemic effect of local IDO protein expression are lacking, except for a study in patients with diffuse large B cell lymphoma, which did not find an association between serum kynurenine level and IDO expression in the tumor (49) and a study in prostate cancer wherein a positive correlation between *IDO1* mRNA in PT samples and the kyn/trp ratio in serum was observed (50).

In this study, for the first time, the association between IDO expression in the tumor microenvironment and systemic concentrations of IDO metabolites in cervical cancer patients was comprehensively investigated, using a validated IDO-specific antibody (51). In the current IHC study, we included a subset of patients from the previously reported serum cohort where association between IDO activity and poor survival was observed (32). Interestingly, we did find increased systemic kyn/trp ratio levels in cervical cancer patients with IDO expression by PT cells rather than IDO expression by immune cells. Moreover, the dominance of tumoral IDO expression on kyn/trp serum levels was independent of the percentage of IDO-positive tumor cells, but rather related to patchy IDO expression, with or without marginal IDO expression (at the tumor/stroma interphase), in the PT. Remarkably, this did not directly impact patient survival. This can be explained by the fact that the current cohort consists of patients with early stage of disease (FFPE material is not available for patients with advanced disease), while in the previous serum study, a patient group comprising various disease stages was analyzed. Interestingly, survival analysis showed that patients with marginal IDO expression in the tumor, including combined patchy + marginal expression, manifested with a significantly improved outcome (DFS: *P* = 0.017; DSS: *P* = 0.043). These data are in concordance with another IDO study in cervical cancer by Inaba and colleagues (30). The marginal IDO effect was proposed to be indicative of an effective IFNγ antitumor T cell response inducing, among others, immunomodulatory factors like PD-L1 and IDO expression in tumor cells (39, 52). In line with this hypothesis, we previously reported on an association between marginal PD-L1 expression and improved prognosis in cervical cancer patients (53). In contrast, and in keeping with our PD-L1 data, patchy IDO expression may result from activation of oncogenic signaling pathways leading to intrinsically elevated expression (4, 54). Interestingly, 7 out of 8 metastatic tumors exhibited patchy IDO expression suggesting that this oncogenic signaling is more pronounced in tumors with an aggressive phenotype and poor patient outcome. Possibly, as indicated by the high kyn/trp serum ratio, IDO expression relating to a patchy expression pattern and putative oncogenic signaling occurs at higher levels than the T cell/IFNγ-induced marginal IDO levels. Although, we did not find higher numbers of (proliferating) T cells in tumors with marginal IDO expression, we did confirm a significant correlation between *IDO1* and *IFNG* mRNA expression by analyzing the available TCGA cervical cancer RNA expression data (37). To draw firm conclusions on the role of IFNγ-producing T cells on IDO expression in the complex tumor microenvironment, more in-depth analysis of the location of these cells and corresponding levels of IFNγ relative to IDO-expressing tumor cells should be performed.

Indoleamine 2,3-dioxygenase has been designated as one of the major immune escape mechanisms employed by tumors. In the cervical tumor microenvironment, IDO positivity was observed in tumor cells, immune cells, and in tumor-associated vessels making it a potential therapeutic target. Although the first clinical results on IDO inhibitors show that they are safe and well-tolerated by patients with different tumor types, no major responses have been observed yet (19, 41, 42, 44). IDO inhibitors are not tested yet in cervical cancer. In contrast to other studies that have shown a correlation between IDO expression and lower cytotoxic T cell infiltration rates and higher frequencies of Tregs, as well as an association of IDO levels with poor prognosis in different tumor types, including colorectal cancer (55), endometrial cancer (56, 57), ovarian cancer (58), and breast cancer (59), our findings did not point to a clear-cut association between IDO protein expression and poor patient outcome.

The finding in the current IHC study rather point to IDO expression in tumor cells and in immune cells as a favorable prognostic factor based on association with disease stage (tumor size and lymph node metastases), survival, and infiltration by actively proliferating cytotoxic T cells. In keeping with this notion, we observed a significant correlation between *IDO1* and *IFNG* mRNA expression, with a survival benefit for patients with high levels of *IFNG*, whether or not combined with high levels of *IDO1* expression. A prognostically favorable association for IDO expression has also previously been observed in breast cancer (22, 24), ovarian cancer (60), renal cell cancer (21), vulvar cancer (61), and lung cancer (51). Notably, in literature, there are contradictory results about the actual effect of tryptophan depletion on proliferating cells (7, 13, 62), and proof is yet lacking for an immunoregulatory role *in vivo* (5). A recent study using 27 cervical cancer punch biopsies showed a correlation between *IDO1* mRNA levels and a high kyn/trp ratio in primary cervical cancer tissue (63), suggesting the presence of functionally active IDO. However, tryptophan depletion *via* IDO might not be efficient enough since tryptophan is able to diffuse rapidly from surrounding tissues into the tumor area (5), or can directly negatively affect the tumor cells themselves (64). This might explain why studies investigating IDO inhibitors in combination with other (immunomodulatory) drugs, like chemotherapy, α-PD-1, and α-CTLA-4, are more promising (43, 65, 66).

This is the first study to apply a multiplex fluorescent immunohistochemistry panel with six different vascular markers for vessel characterization in paraffin-embedded tissue sections. Endothelial IDO expression has previously been reported as an immunoregulatory mechanism in the context of the fetal–maternal interface and of organ transplantation (67–69). In tumors, IDO expression has also been observed in vessels in lymphoma (25, 26), melanoma (70), prostate cancer (50), and renal cell cancer (21). Here, IDO expression was predominantly observed in mature (CD31^+^/CD34^+^/α-sma^+^) tumor-associated blood vessels and in two patients in lymphatic (podoplanin-positive) vessels. IDO-positive vessels were associated with parametrium invasion and higher kyn/trp levels in serum. This is in contrast with another study, which showed IDO to be mainly located in neoangiogenic (CEACAM1-positive) micro-vessels and to correlate with lower rates of tumor cell proliferation (21). However, the number of cases with IDO-positive vessel is small in our study: further analysis on larger cohorts should prove the possible negative effect of IDO-positive vessels on tumor progression.

In conclusion, the effect of IDO in early stage cervical cancer appear to be highly complex. There are several tumor cell expression patterns, many different IDO-positive myeloid cell subtypes as well as varying IDO expression in the vasculature in the tumor microenvironment. Despite this complexity, we have found a dominant effect of patchy IDO expression by PT cells on kyn/trp ratio in serum. Remarkably, marginal IDO expression in tumor fields, independent of the presence of simultaneous patchy IDO expression, was associated with 100% 5-year DSS and DFS. In these patients, the ongoing IFNγ T cell response most likely outweighs any putatively detrimental effect of tryptophan depletion and resulting IDO metabolites. In conclusion, the kyn/trp ratio in serum and *IDO1* mRNA and protein expression *per se* in PTs cannot be used as a clear-cut biomarker for prognosis or to identify early stage cervical cancer patients eligible for clinical trials targeting IDO. Rather, the IDO protein expression patterns in the PT seem vital in this regard.

## Ethics Statement

The specimens were anonymously processed and selection of blocks was guided by initial diagnosis and review by the pathologist. Ethical approval was waived according to the regulations in The Netherlands (http://www.federa.org, 2011).

## Author Contributions

AH performed the experiments, analyzed the data, and wrote the manuscript. ID performed the experiments and analyzed the data. DB and MK performed the experiments. JK and RM performed technical assistance for microscopy. CM, GK, DF and MB contributed to patient inclusion and the final review of the manuscript. VT contributed to data interpretation and to the final review of the manuscript. EJ and TG conceived and designed the experiments, data interpretation, and final review of the manuscript. All authors read and approved the final manuscript.

## Conflict of Interest Statement

The authors declare that the research was conducted in the absence of any commercial or financial relationships that could be construed as a potential conflict of interest.

## Supplementary Material

Figure S1 T cell numbers in relation to IDO expression patterns in primary cervical cancer. Scatter plots show stromal and intratumoral numbers per square millimeters for **(A)** CD8^+^ T cells, **(B)** CD8^+^ T cell/Foxp3^+^ Treg ratio, and **(C)** CD8^+^Ki67^+^ T cells in tumors without IDO-positive tumor-infiltrating immune cells (IDO− infiltrate, white dots, *n* = 14) and with IDO-positive tumor-infiltrating immune cells (IDO+ infiltrate, black squares, *n* = 17). Scatter plot shows **(D)** intratumoral numbers of CD8^+^Ki67^+^ T cells in different groups of primary tumor expression patterns. *P* values were calculated in **(A–C)** using Mann–Whitney *U* test and unpaired *t* test in case of normal distribution of data. *P* values were calculated excluding subgroups with *n* = 2 or smaller (*) in **(D)** using Kruskal–Wallis test. **P* = 0.01–0.05, ***P* = 0.01–0.001, and ****P* < 0.001.
