# Supplementary material for: Indoleamine 2,3-Dioxygenase Expression Pattern in the Tumor Microenvironment Predicts Clinical Outcome in Early Stage Cervical Cancer
Source: Front Immunol. 2018 Jul 11;9:1598. doi: 10.3389/fimmu.2018.01598 (PMC6050387; doi:10.3389/fimmu.2018.01598)
Supplement: Supplementary file 2 [file table_2.docx]

**Supplementary Table 2: IDO protein expression in metastatic lymph nodes.**

IDO+: IDO-positive; IDO-: IDO-negative. *No metastatic tumor cells were present anymore in one stained tissue section. **In three cases we found it difficult to distinguish between IDO+ tumor-infiltrating immune cells and IDO+ tumor cells and excluded those cases for scoring IDO+ infiltrating immune cells. ***In some cases the metastatic tumor involved the whole lymph node and excluded those cases for scoring IDO+ immune cells in peritumoral area and resident lymph node tissue.

|  | **n (%)** |
| --- | --- |
| **Tumor cells**  IDO-  IDO+  Missing*  **IDO expression pattern**  Patchy  Margin  Patchy+margin | 5 (36)  8 (57)  1 (7)  7 (87)  1 (13)  0 (0) |
| **Tumor-infiltrating immune cells**  IDO+  IDO-  Missing** | 9 (64)  2 (14)  3 (22) |
| **IDO+ immune cells in peri-  tumoral area**  High numbers  Low numbers  Missing*** | 12 (86)  1 (7)  1 (7) |
| **IDO+ immune cells in resident  lymph node tissue**  High numbers  Low numbers  Missing*** | 9 (64)  2 (14)  3 (22) |
| **Tumor-associated vessels**  IDO-  IDO+ | 14 (100)  0 (0) |
