# Supplementary figures and images for: Indoleamine 2,3-Dioxygenase Expression Pattern in the Tumor Microenvironment Predicts Clinical Outcome in Early Stage Cervical Cancer
Source: Front Immunol. 2018 Jul 11;9:1598. doi: 10.3389/fimmu.2018.01598 (PMC6050387; doi:10.3389/fimmu.2018.01598)

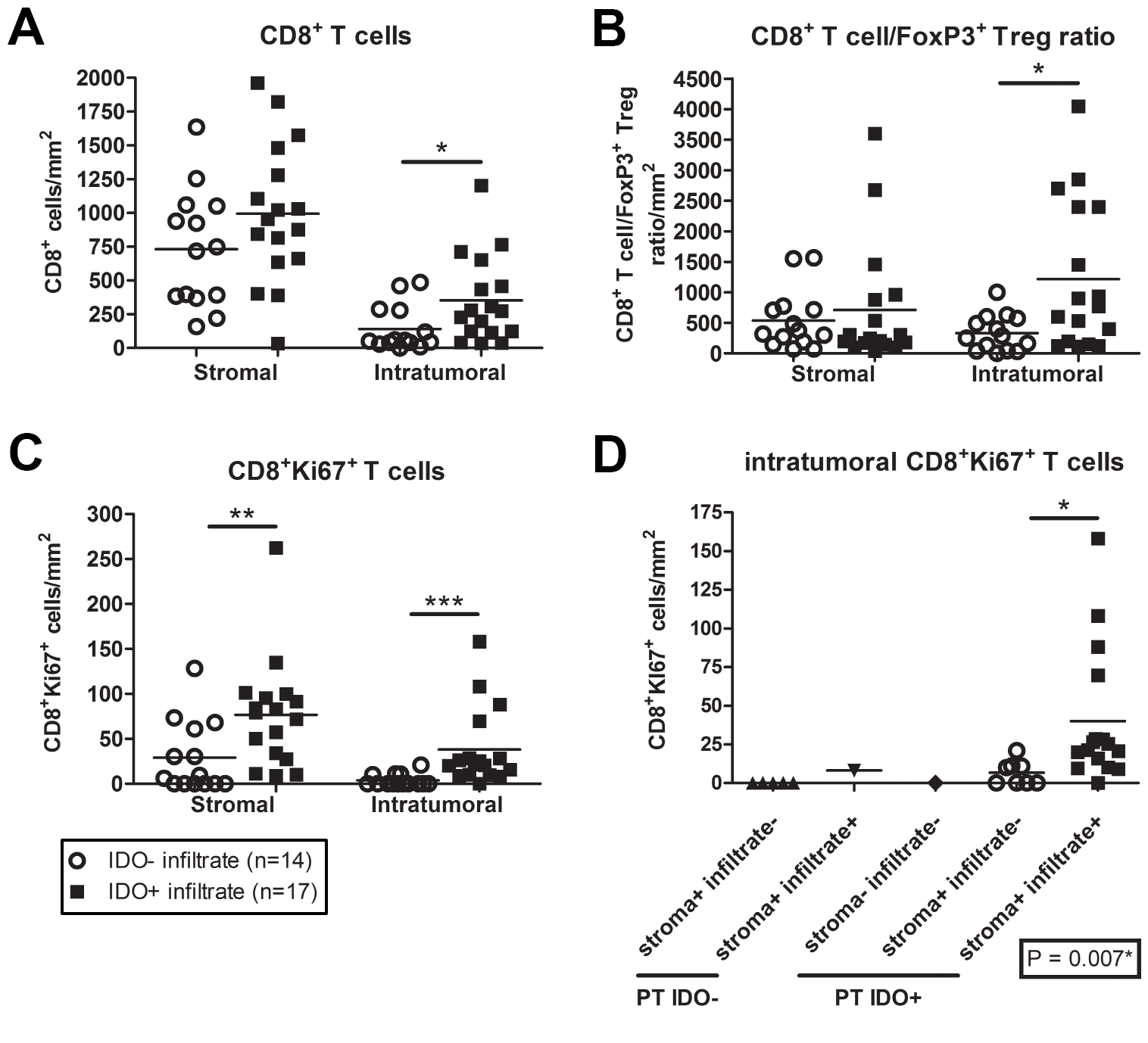

Supplement: Figure S1 — T cell numbers in relation to IDO expression patterns in primary cervical cancer. Scatter plots show stromal and intratumoral numbers per square millimeters for (A) CD8+ T cells, (B) CD8+ T cell/Foxp3+ Treg ratio, and (C) CD8+Ki67+ T cells in tumors without IDO-positive tumor-infiltrating immune cells (IDO− infiltrate, white dots, n = 14) and with IDO-positive tumor-infiltrating immune cells (IDO+ infiltrate, black squares, n = 17). Scatter plot shows (D) intratumoral numbers of CD8+Ki67+ T cells in different groups of primary tumor expression patterns. P values were calculated in (A–C) using Mann–Whitney U test and unpaired t test in case of normal distribution of data. P values were calculated excluding subgroups with n = 2 or smaller (*) in (D) using Kruskal–Wallis test. *P = 0.01–0.05, **P = 0.01–0.001, and ***P < 0.001. [file image_1.tif]
